# Association of psychotropic drug use with falls among older adults in Germany. Results of the German Health Interview and Examination Survey for Adults 2008-2011 (DEGS1)

**DOI:** 10.1371/journal.pone.0182432

**Published:** 2017-08-08

**Authors:** Yong Du, Ingrid-Katharina Wolf, Hildtraud Knopf

**Affiliations:** Department of Epidemiology and Health Monitoring, Robert Koch Institute, Berlin Germany; University of Brescia, ITALY

## Abstract

**Purpose:**

To investigate the association of psychotropic drug use with falls among older adults in Germany based on data from the National Health Interview and Examination Survey for Adults 2008–2011 (DEGS1).

**Methods:**

DEGS1 collected data on drug use in the past 7 days and on falls occurred in the last 12 months. Study participants were older adults aged 65–79 years with complete data on drug use and falls (N = 1,833). Odds ratio (OR) and 95% confidence intervals (95% CI) were derived from logistic regression models adjusting for potential confounders including socio-demographic characteristics, health-related behaviors (alcohol drinking), body mass index and health conditions (frailty, vision impairment, disability, polypharmacy, blood pressure) as well as use of potential falls-risk-increasing drugs. SPSS complex sample methods were used for statistical analysis.

**Results:**

Compared to people without falls, people with falls (n = 370) had a higher psychotropic drug use (33.1% vs. 20.7%, p < .001). After adjusting for potential confounders, use of psychotropic drugs overall was associated with a higher risk of falls (OR 1.64, 95% CI 1.14–2.37). This was particularly true for the use of synthetic psychotropic drugs (1.57, 1.08–2.28), antidepressants overall (2.88, 1.63–5.09) or synthetic antidepressants (2.66, 1.50–4.73), specifically, selective serotonin reuptake inhibitors (SSRIs) (6.22, 2.28–17.0). Similar results were found for recurrent falls.

**Conclusions:**

Use of psychotropic drugs overall, especially synthetic antidepressants like SSRIs, is associated with higher risks of falls and recurrent falls among community dwelling older adults aged 65–79 years in Germany.

## Introduction

As one of the major health problems, falls occur commonly and frequently among older adults with one third of adults aged > = 65 years falling at least once in a given year [1, 2]. Major consequences of falls for individuals include physical injuries and fractures leading to functional decline, disability and decreased quality of life. To society, falls impose high financial burdens and healthcare costs due to fall-caused hospitalization and mortality [3, 4]. In the EU approximately 2.3 million and in the US 2.8 million fall-related injuries are admitted to emergencies while 36,000 adults in the EU and 27,000 older adults in the US are reported to have died from falls each year [2, 5]. The health care expenditure for treating fall-related injuries is estimated to be €25 billion in the EU [5] and $31 billion in the US [6]. In addition, many older adults are afraid of falling, which may result in psychological consequences such as mental stress, depression or anxiety [7].

Falls among older adults are largely preventable by identifying and controlling particularly modifiable risk factors [4, 8, 9]. Use of psychotropic drugs has been identified as an independent risk factor for falls in various studies including systematic reviews and meta-analyses [10–14]. Yet, most of previous studies on psychotropic drug use and falls considered only some of the important health conditions associated with falls such as vision impairment [15, 16], frailty [17], polypharmacy [18], use of potential fall risk-increasing drugs [12, 19] and disability [20]. Results of these studies may be confounded by unmeasured factors. So far, few studies investigating the association between falls and psychotropic drug use have controlled for these factors.

Fall-related injuries among older adults increase along with an aging population [21, 22]. Germany is currently the second oldest population in the world, with 20.9% of the population aged 65 years or over (n = 16.9 million) [23]. About 40% of women and 30% of men aged 65–90 years in Germany report any falls in the past 12 months [24]. Every one in five German adults aged 60–79 years used at least one psychotropic drug in the last 7 days [25]. Since psychotropic drug use is potentially a modifiable factor, further exploring the association of the use of psychotropic drugs, particularly specific subgroups of interest, with falls may provide insight into the prevention strategies of falls among older adults.

Population-based epidemiological studies on the association between psychotropic drug use and falls are lacking in Germany. Based on data of the most recent German Health Interview and Examination Survey for Adults (DEGS1) conducted in 2008–2011, we investigate the use of overall psychotropic drugs, major subgroups of psychotropic drugs as well as specific drugs of interest in relation to any falls and repeated falls after controlling for important health conditions and other factors that are likely to be associated with falls.

## Methods

### Data source: German Health Interview and Examination Survey for Adults 2008–2011 (DEGS1)

The German Health Interview and Examination Survey for Adults, wave 1 (DEGS1) was carried out by the Robert Koch Institute from November 2008 to December 2011 with the aim to provide representative data on the health of adults aged 18–79 years living in Germany [26, 27]. Details of study design, sampling strategy and protocol have been published previously [26, 27]. In Brief, DEGS1 used a two-stage random sampling procedure. First, 180 representative communities (primary sample units, PSU) were randomly sampled from a complete list of German communities proportional to community size. Second, age and sex stratified random samples were drawn within PSUs from local population registries. DEGS1 had a complex design. On one side, DEGS1 invited all surviving participants of the German National Health Interview and Examination Survey 1998 (GNHIES98) in order to establish a survey panel component. On the other side, DEGS1 also recruited new participants in order to maintain a nationally representative sample at the population level [26]. The response rate was 64% for 28–79 year-old re-participants from GNHIES98 (n = 3795) and 42% for newly recruited 18–79 year-old participants (n = 4,192) [26, 27]. Of 3,795 re-participants, 2,923 persons, together with all newly recruited (n = 4,192), completed both the interview and examination survey parts, amounting to a nationally population-representative sample of adults aged 18–79 years (N = 7,115) [26, 27].

DEGS1 was approved by the Federal and State Commissioners for Data Protection and the Charité-Universitätsmedizin Berlin ethics committee (No. EA2/047/08). Survey participants provided written informed consent prior to interviews and examinations.

### Data collection, study population and definition of study variables

Data collection in DEGS1 included self-administered questionnaires, standardized health professional administered computer-assisted personal interviews (CAPI), physiological measurements and laboratory tests [26].

#### Primary outcome: Falls and recurrent falls

In DEGS1, data of falls were collected only among participants 65 years and older. We asked: “*Within the past 12 months*, *have you fallen*, *stumbled or slipped*, *so that you became unbalanced and*, *as a result*, *rested on the floor or lower surface*?” Those who answered with ‘Yes’ were defined as people with falls. Among people with falls, we further asked how many times the falls occurred. Those who answered 2 times or more were defined as people with recurrent falls [24].

#### Primary independent variable: Psychotropic drug use

As part of CAPI, detailed information on drug use (such as daily doses, routes of application and duration of use) in the past 7 days prior to the medical interview was recorded by trained health professionals [28]. In the invitation letter, participants were asked to bring the original packages of all medications used during the past seven days–prescribed and Over-The-Counter (OTC) products- to the examination site for the purpose of documentation and verification. This permitted automated recording of unique product identifiers and drug coding according to the WHO “Anatomical Therapeutic Chemical” (ATC) classification system [28]. Definition of psychotropic drugs has been published previously [25, 29]. Briefly, we included drugs belonging to the nervous system class (ATC code N00) as well as opiates used as antitussives (ATC code R05DA) and aspirin-caffeine combination preparations (ATC code N02BA71) [25, 29] (Appendix Table 1a). We excluded other analgesics and antipyretics such as aspirin and paracetamol (ATC code N02B), local anesthetics (ATC code N01B), homeopathic drugs, and drugs with indistinct active ingredients under the ATC class N00 [25, 29]. Psychotropic drugs with herbal active ingredients were considered and coded separately under specific subgroups (S1 Table).

**Table 1:** Descriptive characteristics of study population. German national health interview and examination survey 2008–2011 (DEGS1).

		Total (N = 1833)				People with falls (n = 370)				People without falls (n = 1463)				p
		%*	95% CI*		n	%*	95% CI*		n	%*	95% CI*		n	
**Sex**	*Men*	46.0	*43*.*5*	*48*.*4*	911	34.2	*28*.*6*	*40*.*3*	142	49.0	*46*.*1*	*52*.*0*	769	**.000**
	*Women*	54.0	*51*.*6*	*56*.*5*	922	65.8	*59*.*7*	*71*.*4*	228	51.0	*48*.*0*	*53*.*9*	694	
**Age group**	*65–69*	34.8	*32*.*3*	*37*.*5*	736	31.4	*25*.*9*	*37*.*4*	139	35.7	*32*.*9*	*38*.*7*	597	.212
	*70–74*	42.8	*39*.*9*	*45*.*9*	744	42.4	*35*.*8*	*49*.*2*	146	43.0	*39*.*7*	*46*.*3*	598	
	*75–79*	22.3	*19*.*8*	*25*.*1*	353	26.2	*21*.*0*	*32*.*3*	85	21.3	*18*.*6*	*24*.*3*	268	
**Living alone**	*Yes*	23.0	*20*.*4*	*25*.*9*	395	32.0	*25*.*5*	*39*.*3*	98	20.7	*18*.*1*	*23*.*5*	297	**.001**
	*No*	77.0	*74*.*1*	*79*.*6*	1434	68.0	*60*.*7*	*74*.*5*	271	79.3	*76*.*5*	*81*.*9*	1163	
**Community size**	*Rural* towns	15.6	*10*.*4*	*22*.*6*	301	14.3	*8*.*7*	*22*.*6*	55	15.9	*10*.*7*	*23*.*0*	246	.404
	*Small cities*	26.4	*19*.*6*	*34*.*5*	430	24.4	*17*.*1*	*33*.*6*	73	26.9	*19*.*9*	*35*.*3*	357	
	*Middle cities*	27.8	*21*.*2*	*35*.*5*	546	27.0	*19*.*6*	*36*.*0*	113	28.0	*21*.*2*	*36*.*0*	433	
	*Large cities*	30.3	*23*.*3*	*38*.*3*	556	34.3	*25*.*9*	*43*.*7*	129	29.2	*22*.*2*	*37*.*4*	427	
**Region of residence**	*Eastern*	23.0	*17*.*3*	*30*.*0*	621	23.1	*16*.*4*	*31*.*6*	119	23.0	*17*.*2*	*30*.*1*	502	.138
	*Northern*	15.8	*10*.*4*	*23*.*3*	236	14.3	*8*.*9*	*22*.*3*	47	16.2	*10*.*6*	*23*.*9*	189	
	*Central*	34.5	*27*.*1*	*42*.*8*	567	39.7	*30*.*8*	*49*.*4*	123	33.2	*25*.*8*	*41*.*5*	444	
	*Southern*	26.7	*19*.*9*	*34*.*7*	409	22.8	*15*.*8*	*31*.*8*	81	27.7	*20*.*5*	*36*.*2*	328	
**Social status**	*Lower*	26.0	*22*.*6*	*29*.*8*	345	24.4	*18*.*1*	*32*.*0*	66	26.5	*22*.*8*	*30*.*5*	279	.767
	*Middle*	59.4	*55*.*8*	*62*.*9*	1105	61.4	*54*.*2*	*68*.*1*	221	58.9	*54*.*7*	*62*.*9*	884	
	*Upper*	14.6	*12*.*5*	*16*.*9*	376	14.2	*10*.*7*	*18*.*7*	80	14.7	*12*.*4*	*17*.*3*	296	
**Body mass index, *kg/m***^***2***^	*<25*	20.3	*18*.*2*	*22*.*6*	400	20.0	*15*.*3*	*25*.*8*	83	20.4	*17*.*9*	*23*.*0*	317	.215
	*25–30*	44.0	*41*.*2*	*46*.*9*	823	39.5	*33*.*6*	*45*.*8*	146	45.2	*41*.*9*	*48*.*5*	677	
	*> = 30*	35.7	*32*.*7*	*38*.*8*	592	40.5	*34*.*2*	*47*.*1*	139	34.4	*31*.*0*	*38*.*0*	453	
**Recognized disability**	*Yes*	28.3	*25*.*2*	*31*.*6*	463	40.6	*33*.*9*	*47*.*7*	127	25.0	*21*.*8*	*28*.*6*	336	**.000**
	*No*	71.7	*68*.*4*	*74*.*8*	1322	59.4	*52*.*3*	*66*.*1*	238	75.0	*71*.*4*	*78*.*2*	1084	
**Polypharmacy**	*Yes*	35.0	*32*.*4*	*37*.*7*	641	39.2	*33*.*2*	*45*.*5*	152	33.9	*31*.*0*	*36*.*9*	489	.115
	*No*	65.0	*62*.*3*	*67*.*6*	1192	60.8	*54*.*5*	*66*.*8*	218	66.1	*63*.*1*	*69*.*0*	974	
**Daily drinking**	*Yes*	18.1	*15*.*7*	*20*.*8*	348	14.9	*10*.*6*	*20*.*4*	63	19.0	*16*.*3*	*22*.*0*	285	.163
	*No*	81.9	*79*.*2*	*84*.*3*	1381	85.1	*79*.*6*	*89*.*4*	287	81.0	*78*.*0*	*83*.*7*	1094	
**Vision impairment**	*No*	77.9	*75*.*2*	*80*.*5*	1393	75.2	*68*.*4*	*81*.*0*	269	78.6	*75*.*3*	*81*.*6*	1124	.179
	*Slight*	18.2	*15*.*8*	*20*.*9*	290	18.5	*13*.*5*	*24*.*8*	61	18.1	*15*.*4*	*21*.*2*	229	
	*Severe*	3.9	*2*.*8*	*5*.*3*	66	6.3	*3*.*4*	*11*.*2*	19	3.3	*2*.*2*	*4*.*7*	47	
**Frailty**	*No*	58.6	*55*.*7*	*61*.*5*	1129	52.8	*45*.*8*	*59*.*6*	201	60.2	*56*.*7*	*63*.*5*	928	**.019**
	*Pre-Frailty*	38.9	*36*.*0*	*41*.*9*	649	42.5	*35*.*7*	*49*.*6*	149	38.0	*34*.*7*	*41*.*4*	500	
	*Frailty*	2.5	*1*.*7*	*3*.*5*	43	4.7	*2*.*7*	*8*.*1*	17	1.9	*1*.*2*	*3*.*0*	26	
**Continuous variables**		Mean*	95% CI*		n	Mean*	95% CI*		n	Mean*	95% CI*		n	
*SBP*, *mm Hg*		130.3	*129*.*2*	*131*.*5*	1828	128.1	*125*.*9*	*130*.*3*	367	130.9	*129*.*7*	*132*.*1*	1461	**.015**
*DBP*, *mm Hg*		72.8	*72*.*2*	*73*.*5*	1828	71.2	*70*.*0*	*72*.*4*	367	73.3	*72*.*5*	*74*.*0*	1461	**.003**

*Weighted and standardized to the population of 31.12.2010. P values: Comparison between people with falls and without falls.

Community size: Rural towns (<5,000 inhabitants), small cities (5,000-<20,000 inhabitants), middle-sized cities (20,000-<100,000 inhabitants) and large cities (100,000 inhabitants or more)

Region of residence: Eastern Germany (Berlin, Brandenburg, Mecklenburg-Vorpommern, Sachsen, Sachsen-Anhalt and Thüringen); Northern Germany (federal states: Bremen, Hamburg, Niedersachsen and Schleswig-Holstein); Central Germany (Hessen, Nordrhein-Westfalen, Rheinland-Pfalz and Saarland); Southern Germany (Baden-Württemberg and Bayern).

Missing values: Living alone (n = 4), social status (n = 7), recognized disability (n = 48), vision impairment (n = 84), daily drinking (n = 104), frailty (n = 12).

For the present analyses, individuals aged 65–79 years with complete data on falls and psychotropic drug use were included as the study population (N = 1,833) (Table 1).

#### Covariables

A number of covariables that are likely to be associated with falls were investigated in this study. This included socio-demographic characteristics (age, sex, community size, region of residence, individual information on household size–namely number of persons living in a household-, income, profession and educational attainment), health-related behaviors, and dietary habits such as alcohol consumption. Information on socio-demographic characteristics, health-related behavior and dietary habits were collected by self-administered questionnaires. Community size was classified as rural towns, small cities, middle-sized cities and large cities based on population density according to an established German classification system. Regions of residence were grouped into 4 commonly described geographical areas: Northern Germany (federal states: *Bremen*, *Hamburg*, *Niedersachsen and Schleswig-Holstein*); Central Germany (*Hessen*, *Nordrhein-Westfalen*, *Rheinland-Pfalz and Saarland*); Southern Germany (*Baden-Württemberg and Bayern*); Eastern Germany (*Berlin*, *Brandenburg*, *Mecklenburg-Vorpommern*, *Sachsen*, *Sachsen-Anhalt and Thüringen*). According to the number of persons living in a household, living alone was defined if only one single person was reported in a household. Socio-economic status (SES) was classified as ‘lower’, ‘middle’ and ‘upper’ using an established index including information on education, professional status and household income [30].

Daily alcohol drinking was assumed if survey participants reported drinking any alcohol-containing beverages at least once a day in the past 12 months [25]. Body mass index (BMI) was calculated as the ratio of body weight (kg) and body height (m) squared and categorized into <25 kg/m^2^, 25–29.9 kg/m^2^ and > = 30 kg/m^2^. Several health conditions were considered in the present analysis based on findings of previous studies: Frailty [17], vision impairment [15, 16], disability [20], polypharmacy [14, 18, 31] and systolic blood pressure (SBP) [32]. Frailty was defined as having 3 and more of the following criteria: Exhaustion, low weight, low physical activity, low walking speed and low grip strength while pre-frailty was defined as having 1–2 of these components [33]. Survey participants were asked if they had any difficulties either in reading printed newspaper or in identifying the face of a person 4 meters away, using glasses or any other reading aid if necessary. Possible answer choices for the question were: (1) can read without difficulty, (2) can read with some difficulty, (3) can read with great difficulty and (4) unable to read at all. Participants in categories ‘3’ or ‘4’ were defined as experiencing ‘severe vision impairment’ and ‘2’ as experiencing ‘some vision impairment’. People were also asked if they had an officially certified disability (yes/no). Further, polypharmacy was assumed if five or more prescription medicines were used in the past 7 days. Three standardized blood pressure (BP) measurements were taken at three minutes intervals in upright sitting with an oscillometric device (Datascope Accutorr Plus). The mean of the second and third measures for systolic BP (SBP) and diastolic BP (DBP) was adopted [34].

A number of drugs have been reported to have the potential to increase the risks of falls (potential fall risk-increasing drugs) [19, 35]. Based on drug use data collected in DEGS1, we considered the major groups of potential falls risk-increasing drugs, namely antihypertensive medications (ATC code C02 antihypertensive, C03 diuretics, C07 beta-adrenergic antagonists, C08 calcium channel blockers and C09 agents acting on the renin-angiotensin system) [36], anti-diabetes medications (ATC code: A10) [19], nonsteroidal anti-inflammatory drugs (NSAIDS) (M01A, M02AA and N02BA) such as aspirin, ibuprofen and naproxen etc. [12, 37], statins (C10AA & C10BA) [38] and thyroid therapy medications (H03) [19].

### Statistical analyses

All statistical analyses were performed using SPSS statistical software (version 20.0, SPSS Inc. Chicago, IL). In order to adjust for sample clustering effects, the SPSS complex samples module was used. Sampling weights were used to correct deviations in the sample from the structure of the German general population of the 31st December 2010 [27].

Participants’ characteristics were summarized by people with falls and without falls in the past 12 months. Differences between people with and without falls were examined using second-order Rao-Scott chi-square tests for categorical variables and using General Linear Models (CSGLM) for continuous variables.

We fitted several logistic models with falls or recurrent fall events in the past 12 months as dependent variables and use of psychotropic drugs as the primary independent variable adjusting risk factors of falls. Model 1 was adjusted for sex and age group. Model 2 was additionally adjusted for social status, community size, region of residence and living alone. Based on model 2, model 3 was further adjusted for health behaviors including daily alcohol drinking, BMI and health conditions including frailty, polypharmacy, disability, vision impairment and SBP. Based on model 3, model 4 was further adjusted for the use of potential falls-risk-increasing dugs including antihypertensives, antidiabetic medications, NSAIDS, statins and thyroid therapy medications. For each of these models, we first looked at the use of overall psychotropic drugs, all synthetic psychotropic drugs and phyto-medicines, and then the use of major subgroups of psychotropic drugs as well as specific drugs or drug classes of interest.

A total of 246 or 13.4% of study participants had missing observations in some variables with a range of 0.2% for living alone, 2.6% for recognized disability, 4.6% for vision impairment, to 5.7% for daily drinking. The numbers of persons with missing values were explicitly stated for each variable (Table 1). Persons with missing values were excluded from the analyses, with pairwise deletion for descriptive and listwise deletion for multivariable analyses (i.e. multivariable models were restricted to participants with valid data on all model covariables). Statistical significance was defined at p<0.05 based on two-sided tests.

## Results

Table 1 shows the descriptive characteristics of the study population stratified by people with and without falls in the past 12 months. Of 1,833 older adults, approximately 20% lived alone or drank daily; more than one third had a BMI > = 30 kg/m^2^ or used polypharmacy; about 40% had frailty or pre-frailty and 22% had a severe or slight vision impairment. 370 older adults reported any falls with a weighted prevalence of 20.7% (95% CI 18.7–23.0%). Of 370 people with falls, 149 (40.3%) reported repeated falls (data not shown in Table 1). Compared to people without falls, people with falls were more likely to be female, to live alone, and to have a recognized disability and a lower average SBP/DBP (Table 1). In addition, they had a higher proportion of frailty and pre-frailty (Table 1). No difference was found between people with and without falls in regard to the distribution of age group, community size, region of residence, social status, BMI, using polypharmacy, daily drinking and vision impairment (Table 1).

Table 2 shows the prevalence for the use of psychotropic drugs as well as potential falls risk-increasing drugs among people with and without falls. People with falls had overall a higher psychotropic drug use compared to people without falls (33.1% vs. 20.7%, p < .001). The same is true for the use of any synthetics (25.1% vs. 16.2%) and phyto-medicines (12.1% vs. 6.3%) as well as the subgroups of anti-depressants, -both synthetic antidepressants (Non-Selective Monoamino Reuptake Inhibitors, NSMRIs and Selective Serotonin Reuptake Inhibitors, SSRIs) and St. John's wort-, and antidementia drugs (mainly Ginkgo biloba) (Table 2). No difference was found between people with and without falls concerning the use of antihypertensives, anti-diabetes medications and statins. Yet, people with falls had a higher use for NSAIDs and thyroid therapy medicines (Table 2).

**Table 2:** Prevalence of psychotropic drug use and potential falls-risk-increasing drug use among older adults with and without falls. German national health interview and examination survey 2008–2011 (DEGS1).

	People with falls (n = 370)				People without falls (n = 1463)				
	%	95%CI		n	%	95%CI		n	p
***Psychotropic drugs***									
Any psychotropic drugs (Synthetics & phytomedicines**)	33.1	*27*.*4*	*39*.*2*	113	20.7	*18*.*0*	*23*.*7*	300	**.000**
Any synthetic psychotropic drugs	25.1	*19*.*9*	*31*.*3*	89	16.2	*13*.*6*	*19*.*1*	231	**.001**
Any phytomedicines	12.1	*7*.*8*	*18*.*1*	33	6.3	*4*.*9*	*8*.*0*	93	**.016**
Any anti-depressants (St. John's wort and synthetical antidepressants)	15.3	*10*.*8*	*21*.*2*	46	6.1	*4*.*6*	*8*.*1*	77	**.000**
*St*. *John's wort* (N05CP03/N06AP01/51(	2.3	.*8*	*6*.*1*	5	.5	.*2*	*1*.*0*	9	**.010**
*Any synthetical antidepressants*	13.0	*8*.*8*	*18*.*6*	41	5.8	*4*.*3*	*7*.*7*	71	**.001**
*NSMRIs(N06AA)*	7.0	*3*.*8*	*12*.*6*	21	3.1	*2*.*1*	*4*.*8*	36	**.019**
*SSRIs (N06AB)*	4.3	*2*.*4*	*7*.*6*	14	1.3	.*8*	*2*.*2*	19	**.002**
Any hypnotics & sedatives (synth., antihistamine and phytoceuticals)	4.3	*2*.*4*	*7*.*5*	19	3.7	*2*.*7*	*5*.*1*	62	.660
*Any synthetics*. *(N05C) and antihistamine (N05CM)*	2.4	*1*.*1*	*5*.*1*	11	1.7	*1*.*1*	*2*.*6*	33	.493
*Any synthetics (N05C)*	2.4	*1*.*1*	*5*.*1*	11	1.3	.*8*	*2*.*1*	25	.207
*Valerian (N05CP01/51)*	1.8	.*7*	*4*.*4*	7	1.6	*1*.*0*	*2*.*6*	27	.887
Any benzodiazepines (N05BA, N05CD, N03AE01)	4.0	*1*.*8*	*8*.*6*	12	2.9	*1*.*9*	*4*.*4*	37	.485
*Benzodiazepine-related drugs (N05CF)*	5.2	*2*.*7*	*9*.*5*	20	3.7	*2*.*6*	*5*.*4*	52	.366
Narcotic analgesics (N02A)	6.6	*4*.*1*	*10*.*5*	23	4.1	*3*.*0*	*5*.*6*	61	.087
Any anti-dementia drugs (N06DA and Ginkgo biloba)	8.3	*4*.*7*	*14*.*2*	24	4.2	*3*.*1*	*5*.*7*	59	**.046**
*Ginkgo biloba (N06DP01)*	8.0	*4*.*4*	*14*.*0*	21	3.9	*2*.*9*	*5*.*3*	55	**.037**
Anti-epileptics (N03)	2.6	*1*.*3*	*5*.*0*	13	1.9	*1*.*0*	*3*.*3*	30	.448
Antiparkinson drugs (N04)	2.5	*1*.*1*	*5*.*5*	9	1.4	.*7*	*2*.*6*	21	.203
***Potential falls-risk-increasing dugs***									
Any antihypertensives (ATC-Code C02, C03, C07, C08, C09)	65.5	*59*.*1*	*71*.*4*	246	68.9	*65*.*8*	*71*.*8*	984	.307
Anti-diabetes medications (A10)	17.0	*12*.*6*	*22*.*5*	58	13.6	*11*.*3*	*16*.*3*	196	.193
**NSAIDs (M01A, M02AA and N02BA)**	**29.1**	***23*.*9***	***34*.*9***	**107**	**19.0**	***16*.*1***	***22*.*2***	**260**	**.000**
Statins (C10AA & C10BA)	25.4	*20*.*6*	*30*.*8*	106	27.0	*24*.*3*	*29*.*8*	399	.596
Thyroid therapy (H02)	24.2	*18*.*6*	*30*.*9*	86	17.6	*14*.*9*	*20*.*6*	253	**.031**

* Weighted and standardized to the population of 31.12.2010.

P-values: Comparison between people with falls and people without falls

**Phytomedicines: *St*. *John's wort (N06AP)*, Ginkgo biloba (N06DP01), *Valerian (N05CP)*.

Figures in bold denote statistical significance (p < .050)

NSMRIs: Non-Selective Monoamino Reuptake Inhibitors

SSRIs: Selective Serotonin Reuptake Inhibitors

NSAIDs: Nonsteroidal Anti-Inflammatory Drugs (M01A, M02AA and N02BA)

In logistic regression models adjusting for age and sex, use of overall psychotropic drugs and of synthetic psychotropic drugs was associated with falls, but this did not apply to the use of phytomedicines (Table 3). Further adjusting for confounders included in model 2, model 3 and model 4, the association remains robust (Table 3). The risk of any falls was 64% higher among psychotropic drug users compared to nonusers. In terms of major subgroups of psychotropic drugs, use of antidepressants overall increased the risk of falls significantly after considering all potential confounders (OR 2.88, 95% CI 1.63–5.09). Use of synthetic antidepressants (2.66, 1.50–4.73), but not St. John’s wort was associated with any falls. Specifically, use of SSRIs (6.22, 2.28–17.0), but not non-selective monoamine reuptake inhibitors (NSMRIs) increased the risk of any falls (Table 3). For the use of other psychotropic drugs, no significantly increased risks were found (Table 3).

**Table 3:** Association of psychotropic drug use with risks of falls. German national health interview and examination survey 2008–2011 (DEGS1).

	Model 1			Model 2			Model 3			Model 4		
	OR	95%CI		OR	*95%CI*		OR	95%CI		OR	95%CI	
Any psychotropic drugs (Synthetics & phytomedicines)	**1.69**	***1*.*24***	***2*.*31***	**1.68**	***1*.*23***	***2*.*29***	**1.64**	***1*.*14***	***2*.*35***	**1.64**	***1*.*14***	***2*.*37***
Any synthetic psychotropic drugs	**1.58**	***1*.*12***	***2*.*24***	**1.61**	***1*.*14***	***2*.*29***	**1.52**	***1*.*05***	***2*.*21***	**1.57**	***1*.*08***	***2*.*28***
Any phytomedicines	1.76	*0*.*98*	*3*.*19*	1.62	*0*.*91*	*2*.*89*	1.66	*0*.*84*	*3*.*29*	1.62	*0*.*82*	*3*.*19*
Any anti-depressants (St. John's wort and synthetical antidepressants)	**2.39**	***1*.*43***	***4*.*01***	**2.33**	***1*.*39***	***3*.*90***	**2.69**	***1*.*50***	***4*.*83***	**2.88**	***1*.*63***	***5*.*09***
*St*. *John's wort* (N05CP03/N06AP01/51(	**4.36**	***1*.*30***	***14*.*6***	**4.66**	***1*.*21***	***18*.*0***	4.22	*0*.*62*	*28*.*9*	3.71	*0*.*64*	*21*.*6*
*Any synthetical antidepressants*	**2.10**	***1*.*21***	***3*.*62***	**2.01**	***1*.*16***	***3*.*47***	**2.43**	***1*.*36***	***4*.*34***	**2.66**	***1*.*50***	***4*.*73***
*NSMRIs(N06AA)*	1.95	*0*.*89*	*4*.*24*	1.72	*0*.*80*	*3*.*68*	1.75	*0*.*77*	*4*.*01*	1.84	*0*.*83*	*4*.*10*
*SSRIs (N06AB)*	**2.97**	***1*.*31***	***6*.*74***	**3.18**	***1*.*34***	***7*.*56***	**5.46**	***1*.*96***	***15*.*2***	**6.22**	***2*.*28***	***17*.*0***
Any hypnotics & sedatives (synth., antihistamine and phytoceuticals)	0.99	*0*.*49*	*2*.*00*	1.05	*0*.*52*	*2*.*13*	0.88	*0*.*42*	*1*.*83*	0.79	*0*.*37*	*1*.*71*
Any synth. (N05C) and antihistamine (N05CM)	1.27	*0*.*53*	*3*.*03*	1.30	*0*.*53*	*3*.*18*	0.67	*0*.*28*	*1*.*61*	0.58	*0*.*22*	*1*.*49*
Any benzodiazepines (N05BA, N05CD, N03AE01)	1.20	*0*.*47*	*3*.*07*	1.22	*0*.*48*	*3*.*14*	0.90	*0*.*24*	*3*.*39*	0.90	*0*.*25*	*3*.*28*
Benzodiazepines and benzodiazepine-related drugs (N05CF)	1.22	*0*.*57*	*2*.*60*	1.24	*0*.*58*	*2*.*66*	0.89	*0*.*32*	*2*.*44*	0.85	*0*.*31*	*2*.*32*
Narcotic analgesics (N02A)	1.52	*0*.*84*	*2*.*72*	1.58	*0*.*87*	*2*.*88*	1.39	*0*.*68*	*2*.*84*	1.48	*0*.*70*	*3*.*12*
Any anti-dementia drugs (N06DA+ Ginkgo biloba)	1.81	*0*.*90*	*3*.*68*	1.53	*0*.*78*	*3*.*03*	1.60	*0*.*71*	*3*.*64*	1.57	*0*.*69*	*3*.*60*
Ginkgo biloba (N06DP01)	1.87	*0*.*91*	*3*.*85*	1.56	*0*.*77*	*3*.*14*	1.64	*0*.*71*	*3*.*77*	1.61	*0*.*69*	*3*.*74*
Anti-epileptics (N03)	1.58	*0*.*62*	*3*.*98*	1.76	*0*.*75*	*4*.*17*	1.36	*0*.*51*	*3*.*61*	1.47	*0*.*58*	*3*.*73*
Antiparkinson drugs (N04)	1.76	*0*.*70*	*4*.*40*	1.80	*0*.*70*	*4*.*65*	1.12	*0*.*31*	*4*.*08*	1.08	*0*.*26*	*4*.*55*
Valerian (N05CP01/51)	0.73	*0*.*26*	*2*.*05*	0.79	*0*.*28*	*2*.*21*	0.96	*0*.*33*	*2*.*77*	0.90	*0*.*31*	*2*.*61*

Odds ratio (OR) and 95% confidence intervals (95% CI) were derived from logistic regression models.

Model 1: adjusted for sex and age group (65–69, 70–74, 75–79 years)

Model 2: model 1+social status (lower, middle, upper), community size (rural area, small, middle and large city), region (East, north, central, south), living alone (yes, no),

Model 3: Model 2+ frailty (frailty, pre-frailty, no frailty), polypharmacy (yes, no), disability (yes, no), vision impairment (severe, slight, no), daily alcohol drinking (yes, no), BMI <25, 25–30, > = 30), SBP (continuous variables)

Model 4: Model 3+antihypertensives, antidiabetics, nonsteroidal anti-inflammatory drugs (NSAIDs), statins, thyroid therapy (yes, no)

NSMRIs: non-selective monoamino reuptake inhibitors

SSRIs: Selective serotonin reuptake inhibitors

Figures in bold denote statistical significance (p < .050)

As with falls, use of overall psychotropic drugs was also associated with recurrent falls (adjusted OR 1.84, 95% CI 1.02–3.31) (Table 4). The odds ratios for the relationship between recurrent falls and the use of overall antidepressants, synthetic antidepressants, such as SSRIs, were consistently significant (p<0.05) in all 4 models. The odds ratios for narcotic analgesics (N02A) and anti-Parkinson drugs (N04) were found to be significant in model 1 and model 2, but not in model 3 and model 4 (Table 4).

**Table 4:** Association of psychotropic drug use and risk of recurrent falls. German national health interview and examination survey 2008–2011 (DEGS1).

	Model 1			Model 2			Model 3			Model 4		
	OR	95%CI		OR	95%CI		OR	95%CI		OR	95%CI	
Any psychotropic drugs (Synthetics & phytomedicines)	**1.97**	***1*.*27***	***3*.*05***	**1.96**	***1*.*25***	***3*.*08***	1.83	*0*.*99*	*3*.*35*	**1.84**	***1*.*02***	***3*.*31***
Any synthetics	**1.65**	***1*.*04***	***2*.*64***	**1.70**	***1*.*05***	***2*.*75***	1.30	*0*.*77*	*2*.*20*	1.38	*0*.*83*	*2*.*29*
Any phytomedicines	1.60	*0*.*74*	*3*.*44*	1.44	*0*.*64*	*3*.*24*	1.76	*0*.*58*	*5*.*35*	1.69	*0*.*57*	*5*.*00*
Any anti-depressants (St. John's wort and all synthetical antidepressants)	**2.09**	***1*.*14***	***3*.*82***	**2.08**	***1*.*10***	***3*.*95***	**2.74**	***1*.*36***	***5*.*51***	**3.15**	***1*.*60***	***6*.*23***
*St*. *John's wort* (N05CP03/N06AP01/51(	1.10	*0*.*22*	*5*.*58*	1.19	*0*.*20*	*7*.*16*	3.30	*0*.*46*	*23*.*7*	2.76	*0*.*30*	*25*.*3*
Any *synthetical antidepressants*	**2.16**	***1*.*15***	***4*.*07***	**2.12**	***1*.*08***	***4*.*15***	**2.53**	***1*.*22***	***5*.*27***	**3.01**	***1*.*48***	***6*.*09***
*NSMRIs(N06AA)*	1.28	*0*.*51*	*3*.*23*	1.11	*0*.*40*	*3*.*06*	0.95	*0*.*35*	*2*.*57*	1.11	*0*.*42*	*2*.*92*
*SSRIs (N06AB)*	**3.10**	***1*.*25***	***7*.*69***	**3.55**	***1*.*42***	***8*.*85***	**6.74**	***2*.*47***	***18*.*4***	**7.02**	***2*.*38***	***20*.*7***
Any hypnotics & sedatives (synth., antihistamine and phytoceuticals)	0.74	*0*.*27*	*2*.*08*	0.80	*0*.*29*	*2*.*22*	0.89	*0*.*28*	*2*.*88*	0.72	*0*.*22*	*2*.*40*
Any synth. (N05C) and antihistamine (N05CM)	0.73	*0*.*20*	*2*.*65*	0.81	*0*.*21*	*3*.*08*	0.56	*0*.*13*	*2*.*51*	0.45	*0*.*10*	*2*.*08*
Benzodiazepines (N05BA, N05CD, N03AE01)	1.23	*0*.*40*	*3*.*78*	1.28	*0*.*42*	*3*.*92*	0.93	*0*.*16*	*5*.*22*	1.01	*0*.*17*	*5*.*88*
Benzodiazepines and benzodiazepine-related drugs	1.31	*0*.*52*	*3*.*26*	1.41	*0*.*57*	*3*.*49*	0.92	*0*.*24*	*3*.*48*	0.88	*0*.*22*	*3*.*56*
Narcotic analgesics (N02A)	**2.64**	***1*.*30***	***5*.*38***	**2.66**	***1*.*30***	***5*.*43***	2.21	*0*.*97*	*5*.*05*	2.27	*0*.*97*	*5*.*34*
Any anti-dementia drugs (N06DA+ Ginkgo biloba)	2.21	*0*.*95*	*5*.*11*	1.85	*0*.*73*	*4*.*64*	1.91	*0*.*50*	*7*.*26*	1.92	*0*.*54*	*6*.*79*
Ginkgo biloba (N06DP01)	2.15	*0*.*89*	*5*.*20*	1.78	*0*.*67*	*4*.*76*	1.85	*0*.*45*	*7*.*56*	1.90	*0*.*51*	*7*.*09*
Anti-epileptics (N03)	0.74	*0*.*21*	*2*.*59*	0.85	*0*.*24*	*2*.*94*	0.47	*0*.*11*	*1*.*99*	0.42	*0*.*10*	*1*.*81*
Antiparkinson drugs (N04)	**3.51**	***1*.*18***	***10*.*4***	**3.80**	***1*.*29***	***11*.*2***	1.44	*0*.*34*	*6*.*02*	1.43	*0*.*37*	*5*.*56*
Valerian (N05CP01/51)	0.71	*0*.*16*	*3*.*23*	0.76	*0*.*17*	*3*.*35*	1.10	*0*.*21*	*5*.*78*	0.89	*0*.*16*	*4*.*80*

Odds ratio (OR) and 95% confidence intervals (95% CI) were derived from logistic regression models.

Model 1: adjusted for sex and age group (65–69, 70–74, 75–79 years)

Model 2: model 1+social status (lower, middle, upper), community size (rural area, small, middle and large city), region (East, north, central, south), living alone (yes, no),

Model 3: Model 2+ frailty (frailty, pre-frailty, no frailty), polypharmacy (yes, no), disability (yes, no), vision impairment (severe, slight, no), daily alcohol drinking (yes, no), BMI <25, 25–30, > = 30), SBP (continuous variables)

Model 4: Model 3+antihypertensives, antidiabetics, nonsteroidal anti-inflammatory drugs (NSAIDs), statins, thyroid therapy (yes, no)

NSMRIs: non selective monoamino reuptake inhibitors

SSRIs: Selective serotonin reuptake inhibitors

Figures in bold denote statistical significance (p < .050).

Sensitivity analyses involved limiting to those who had used any psychotropic drugs over 12 months and results were similar. In the fully adjusted Model 4, the OR for any psychotropic drug use over 12 months was 1.70 (95% CI 1.10–2.63) for any falls and 2.10 (95% CI 1.04–4.22) for recurrent falls; the OR for any synthetic drug use over 12 months was 1.89 (95% CI 1.21–2.94) for any falls and 1.92 (95% CI 1.11–3.34) for recurrent falls (data not shown in Tables 3 and 4).

## Discussion

### Main findings

In the present study, we found that the use of psychotropic drugs overall was associated with increased risks of falls and recurrent falls in the past 12 months among community-dwelling adults aged 65–79 years in Germany. This is particularly true for the use of synthetic psychotropic drugs, mainly synthetic antidepressants, -specifically SSRIs. Our study confirms the association between psychotropic drug use and falls and adds evidence for an increased risk of falls following the use of psychotropic drugs overall and specific subgroups among community-dwelling older adults.

### Comparison with other studies

The association between psychotropic drug use and falls has been investigated in previous studies and systematically reviewed or meta-analyzed [10–14]. An earlier meta-analysis covering studies published between 1966–1996 examining the association of drug use with falling in people aged 60 and older found a pooled odds ratio of 1.73 (95%CI, 1.52–1.97) for any psychotropic use [10]. This finding was confirmed by another meta-analysis including 71 studies published between 1981 and 2007, which reported an odds ratio of 1.78 (1.57–2.01) [13]. Both are well comparable to our finding of an odds ratio of 1.64 (1.14–2.37) for the association between psychotropic drug use and falls among older adults. A recent systematical review of prospective and retrospective studies published between 2008 and 2013 on the association of medication use and falls in older people found that 29 of 36 studies reported a positive association between the risk of falls and use of psychotropic medications including sedatives and hypnotics, antidepressants, and benzodiazepines [14]. Further, a large body of evidence from prospective studies and clinical trials suggests that reduced use of psychotropic medications resulted in reduction of falls [39], strongly supporting the associations between psychotropic drug use and falls.

Our findings on the association between antidepressants use and falls or recurrent falls are also in line with results of other studies. Use of antidepressants, particularly SSRIs, has been consistently found to be associated with increased risks of falls and recurrent falls [40–43]. For example, in a longitudinal analysis of 2948 community-dwelling older adults followed-up for 7 years, antidepressant users, compared with nonusers, were observed to have a 48% greater likelihood of recurrent falls (OR 1.48, 1.12–1.96), particularly those taking SSRIs with an OR of 1.62 (1.15–2.28) [40]. Alike, in a cross-sectional survey including community-dwelling older adults from Australian general practices, antidepressants use was independently associated with multiple falls (OR 1.46, 1.25–1.70). Amongst all psychotropic medications, SSRI use was found to be associated with the highest risk of multiple falls (OR 1.66, 1.36–2.02) [44]. Using data of the Swedish registers with a sample size of more than 1 million persons aged ≥65 years, Johnell and colleagues found that among psychotropic drugs, antidepressants had the strongest association with fall-related injuries (adjusted OR 1.42, 1.38–1.45) [45]. These studies suggest that use of antidepressants could result in a 40–70% greater likelihood of falls [40, 44, 45]. In the present study, we found a much higher OR of 2.88 (1.63–5.09) for the association between use of antidepressants and falls, which, however, is comparable to the results of a prospective population-based study reporting an OR of 2.8 (1.9–4.1) among older men aged 60–75 years in Denmark [46]. Nevertheless due to small sample size in our study, the OR for SSRIs use (Table 3) is much higher than that (3.1, 2.0–5.0) found in the Danish study [46].

Interestingly, we found that the use of psychotropic phyto-medicines, either St. Johns’ wort, valerian or Gingko biloba, was not associated with any falls or recurrent falls. This may be due to their weak effects compared to their synthetic counterparts. Surprisingly, we did not find an association of falls or recurrent falls with the use of hypnotics and sedatives, antiepileptics, or narcotic analgesics. However, some studies also found no significant association between falls and the use of anxiolytics/hypnotics [46], or a weaker association with the use of hypnotics and sedatives [45] or antiepilepitcs [47]. In contrast, other studies report positive associations between falls and the use hypnotics and sedatives [12, 14, 47, 48], opioids/narcotics [46, 48][49] or antiepileptics [46]. There may be several reasons for the inconsistence between findings of our study and of others. First, the sample size for users of hypnotics and sedatives, antiepileptics and narcotic analgesics in our study is relatively small (for example, only 36 persons used synthetic hypnotics and sedatives, Table 2). Second, more than half of all benzodiazepines, hypnotics and sedatives were taken as needed while only a small part of them were taken regularly more than 3 months (appendix Table 1a). Third, evidence of an association between substantially increased risk of falls and use of benzodiazepines (particularly long-acting agents) tends to be found in earlier study. Finally, because of well-known ‘after effects’ of hypnotics and sedatives that may increase the risk of falls, older adults at high risk of falls may have been cautioned by physicians and thus avoided taking such kind of drugs. As a result, use of anxiolytics was found to be even associated with a slightly reduced risk of fall (OR 0.97, 95% CI 0.94–1.00) [45]. In our study, although not statistically significant, the adjusted ORs for the use of synthetic hypnotics & sedatives, benzodiazepine and related drugs tend to be <1 (Table 3).

### Strength and limitation

The main strength of our study is that we used a nationally representative sample of community-dwelling older adults. The weighted results can be generalized to the German older adults aged 65–79 years. We fitted several logistic regression models to explore the associations between psychotropic drug use and falls controlling for a number of confounding variables including frailty, vision impairment and use of other fall risk-increasing drugs.

Findings of our study are subject to several limitations. First, DEGS1 is not specifically designed for the study of association of psychotropic drug use and falls. Since other sources of linked health administrative data are unavailable, data of national health surveys are a good source that could be used for such kind of studies despite limitations. Persons aged > = 80 years and persons who were hospitalized or institutionalized in long-term care facilities were not included in our national health surveys. In addition, community-dwelling older adults with cognitive impairment, depression or other severe mental or physical illnesses (e.g. severe vision impairment and fall injuries) might be less likely to take part in the survey due to the need to travel to the examination sites. These persons might be at psychotropic drug use and high risk of falls. Second, data on falls and drug use were self-reported; recall bias seems unavoidable. In the invitation letter we asked all survey participants to bring the medication packages to examination sites allowing us to verify medication use, which could reduce recall bias greatly. Third, findings of our study might be weakened by the fact that we measured falls in the past 12 months whereas drug use in the last 7 days. Due to this concern, we therefore specifically examined use pattern and use durations of all psychotropic drugs. Results reveal that more than half of all psychotropic drugs have been used for 12 months or longer. This is particularly true for the use of major subgroups of psychotropic drugs such as synthetic antidepressants (72.4%) (S2 Table). Furthermore, sensitivity analyses involving drug use over 12 months demonstrate similar results, further supporting our findings. Fourth, the number of drug users in some subgroups was quite small, which prevented us from detecting weak associations. Fifth, we investigated if any specific psychotropic drug was used in relations to falls. Nevertheless, total daily drug dose exposure, changing doses, interactions between drugs, can all be involved in fall risks, which we could not investigate in the present analysis. Finally and most importantly, subject to observational design, our study did not allow a causality inference between falls and psychotropic drug use and could not avoid indication bias. Indication bias occurs when patients are prescribed drugs for a condition that itself is associated with the outcome of interest [50, 51]. In this analysis, antidepressants are indicated to treat depression, which itself, has been recognized as a key independent risk factor for falls [52–54]. Yet, there are also studies suggesting that antidepressant use (particularly SSRIs) is strongly associated with falls regardless of the presence of depressive symptoms [44].

## Conclusions

In summary, we found that the use of psychotropic drugs overall, especially synthetic antidepressants like SSRIs, was associated with a higher risk of falls and recurrent falls among community dwelling older adults aged 65–79 years in Germany. Our study adds new evidence concerning the association of use of psychotropic drugs—mainly synthetic antidepressants like SSRIs- and falls among community-dwelling older adults. Given the severe consequence of falls and the extensive use of psychotropic drugs among the elderly, a more rational use of such kind of drugs is needed. Due to limitations of our study with small sample sizes for some subgroups of drugs, further studies are required to investigate the associations between the use of other commonly used psychotropic drugs such as hypnotics and sedatives and falls.

## Supporting information

S1 Table List of psychotropic drugs used among people aged 65–79 years. German national health interview and examination survey 2008–2011 (DEGS1).
(DOCX)

S2 Table Use pattern of psychotropic drugs among people aged 65–79 years—by use duration. German national health interview and examination survey 2008–2011 (DEGS1).
(DOCX)
