# Supplementary material for: Association of psychotropic drug use with falls among older adults in Germany. Results of the German Health Interview and Examination Survey for Adults 2008-2011 (DEGS1)
Source: PLoS One. 2017 Aug 8;12(8):e0182432. doi: 10.1371/journal.pone.0182432 (PMC5549725; doi:10.1371/journal.pone.0182432)
Supplement: S1 Table — German national health interview and examination survey 2008–2011 (DEGS1). (DOCX) [file pone.0182432.s001.docx]

**S1 Table**

**S1 Table. List of psychotropic drugs used among people aged 65-79 years. German national health interview and examination survey 2008-2011 (DEGS1)**

| **Atc-code** | **Ingredients** | **Frequency** |
| --- | --- | --- |
| N02AA01 | Morphine | 3 |
| N02AA03 | Hydromorphone | 3 |
| N02AA05 | Oxycodone | 4 |
| N02AA55 | Oxycodone and naloxone | 1 |
| N02AA65 | Codeine and diclofenac | 1 |
| N02AA69 | Codeine and paracetamol | 7 |
| N02AB03 | Fentanyl | 7 |
| N02AE01 | Buprenorphine | 6 |
| N02AX02 | Tramadol | 21 |
| N02AX51 | Tilidin, combinations | 28 |
| N02AX52 | Tramadol, combinations | 3 |
| N02CA01 | Dihydroergotamine | 1 |
| N02CC01 | Sumatriptan | 2 |
| N02CC03 | Zolmitriptan | 1 |
| N02CC07 | Frovatriptan | 1 |
| N03AA03 | Primidone | 1 |
| N03AE01 | Clonazepam | 1 |
| N03AF01 | Carbamazepine | 14 |
| N03AF02 | Oxcarbazepine | 2 |
| N03AG01 | Valproic acid | 6 |
| N03AX09 | Lamotrigine | 1 |
| N03AX12 | Gabapentin | 12 |
| N03AX16 | Pregabalin | 12 |
| N04AA11 | Bornaprine | 2 |
| N04BA03 | Levodopa, decarboxylase inhibitor and COMT inhibitor | 1 |
| N04BA10 | Levodopa in combination with carbidopa | 10 |
| N04BA11 | Levodopa in Kombination mit Benserazid | 17 |
| N04BB01 | Amantadine | 1 |
| N04BC04 | Ropinirole | 2 |
| N04BC05 | Pramipexole | 11 |
| N04BC09 | Rotigotine | 1 |
| N05AA02 | Levomepromazine | 1 |
| N05AC02 | Thioridazine | 1 |
| N05AD03 | Melperone | 1 |
| N05AH03 | Olanzapine | 2 |
| N05AH04 | Quetiapine | 2 |
| N05AL01 | Sulpiride | 5 |
| N05AN01 | Lithium | 4 |
| N05AX11 | Zotepine | 1 |
| N05BA01 | Diazepam | 5 |
| N05BA03 | Medazepam | 1 |
| N05BA04 | Oxazepam | 5 |
| N05BA05 | Potassium clorazepate | 2 |
| N05BA06 | Lorazepam | 8 |
| N05BA08 | Bbomazepam | 12 |
| N05BA11 | Prazepam | 2 |
| N05BA12 | Alprazolam | 3 |
| N05BB01 | Hydroxyzine | 1 |
| N05CD02 | Nitrazepam | 4 |
| N05CD03 | Flunitrazepam | 1 |
| N05CD06 | Lormetazepam | 4 |
| N05CD09 | Brotizolam | 2 |
| N05CF01 | Zopiclone | 9 |
| N05CF02 | Zolpidem | 14 |
| N05CH01 | Melatonin | 2 |
| N05CM21 | Doxylamine | 5 |
| N05CM22 | Promethazine | 3 |
| N05CP01 | Valerianae radix | 24 |
| N05CP03 | St. John's wort | 3 |
| N05CP04 | Herbs of melissa | 6 |
| N05CP05 | Herbs of passion flower | 1 |
| N05CP51 | Valerianae radix, combinations | 12 |
| N06AA04 | Clomipramine | 1 |
| N06AA05 | Opipramol | 16 |
| N06AA06 | Trimipramine | 7 |
| N06AA09 | Amitryptiline | 18 |
| N06AA12 | Doxepin | 17 |
| N06AA21 | Maprotiline | 1 |
| N06AB03 | Fluoxetine | 3 |
| N06AB04 | Citalopram | 23 |
| N06AB05 | Paroxetine | 2 |
| N06AB06 | Sertraline | 2 |
| N06AB10 | Escitalopram | 3 |
| N06AP01 | St. John's wort, | 9 |
| N06AP51 | St. John's wort, combinations | 2 |
| N06AX11 | Mirtazapine | 18 |
| N06AX16 | Venlafaxine | 9 |
| N06AX21 | Duloxetine | 7 |
| N06AX22 | Agomelatine | 1 |
| N06BX03 | Piracetam | 4 |
| N06DA02 | Donepezil | 5 |
| N06DA03 | Rivastigmine | 1 |
| N06DA04 | Galantamine | 1 |
| N06DP01 | Dry extract of gingko leaves | 77 |
| N06DX13 | Nicergoline | 1 |
| N07AA02 | Pyridostigmine | 1 |
| N07AA03 | Distigmine | 1 |
| N07AX01 | Pilocarpine | 1 |
| N07CA01 | Betahistine | 9 |
| N07CA52 | Cinnarizine, combinations | 7 |
| N07XB01 | Thioctid acid | 1 |
| N07XB52 | Thiamine, combinations | 1 |
| N07XB56 | Benfotiamine, combinations | 2 |
| R05DA04 | Codeine | 5 |
| R05DA09 | Dextromethorphan | 1 |
| R05DA14 | Dihydrocodein | 1 |
