# Supplementary material for: Association of psychotropic drug use with falls among older adults in Germany. Results of the German Health Interview and Examination Survey for Adults 2008-2011 (DEGS1)
Source: PLoS One. 2017 Aug 8;12(8):e0182432. doi: 10.1371/journal.pone.0182432 (PMC5549725; doi:10.1371/journal.pone.0182432)
Supplement: S2 Table — German national health interview and examination survey 2008–2011 (DEGS1). (DOCX) [file pone.0182432.s002.docx]

**S2 Table**

**S2 Table. Use pattern of psychotropic drugs among people aged 65-79 years – by use duration. German national health interview and examination survey 2008-2011 (DEGS1)**

|  |  | As needed | | <3 Months | | 3-12 months | | >=12 months | |
| --- | --- | --- | --- | --- | --- | --- | --- | --- | --- |
|  | N (%) | *n* | % | *n* | % | n | % | n | % |
| **Synthetics** |  |  |  |  |  |  |  |  |  |
| Antidepressants (N06A, incl, N06AB, N06AA, N06AX02-22) | 127(100) | *9* | 7.1 | *5* | 3.9 | *21* | 16.5 | *92* | 72.4 |
| Opioids (N02A & R05DA) | 91(100) | *34* | 37.4 | *11* | 12.1 | *17* | 18.7 | *29* | 31.9 |
| Anti-migraine medications (N02C) | 5(100) | *4* | 80.0 | *0* | 0.0 | *0* | 0.0 | *1* | 20.0 |
| Antiepileptics (N03) | 49(100) | *3* | 6.1 | *1* | 2.0 | *7* | 14.3 | *38* | 77.6 |
| Antiparkinsonian agents (N04) | 45(100) | *4* | 8.9 | *4* | 8.9 | *9* | 20.0 | *28* | 62.2 |
| Antipsychotics (N05A) | 17(100) | *1* | 5.9 | *0* | 0.0 | *0* | 0.0 | *16* | 94.1 |
| Anxiolytics (N05B) | 38(100) | *23* | 60.5 | *2* | 5.3 | *1* | 2.6 | *12* | 31.6 |
| Hypnotics and sedatives (N05C) | 36(100) | *16* | 44.4 | *0* | 0.0 | *1* | 2.8 | *19* | 52.8 |
| Antihistamine agents as hypnotics and sedatives (N05CM21/22) | 8(100) | *7* | 87.5 | *0* | 0.0 | *1* | 12.5 | *0* | 0.0 |
| Anti-dementia drugs (N06DA02/04) | 7(100) | *0* | 0.0 | *2* | 28.6 | *2* | 28.6 | *3* | 42.9 |
| Psychostimulants and nootropics (N06B) | 4(100) | *0* | 0.0 | *1* | 25.0 | *0* | 0.0 | *3* | 75.0 |
| Antivertigo preparations & others (N07CA, N07CH20, N07XX) | 27(100) | *2* | 7.4 | *4* | 14.8 | *1* | 3.7 | *20* | 74.1 |
| **All synthetics** | **454(100)** | ***103*** | **22.7** | ***30*** | **6.6** | ***60*** | **13.2** | ***261*** | **57.5** |
|  |  |  |  |  |  |  |  |  |  |
| **Phytomedicines** |  |  |  |  |  |  |  |  |  |
| Gingko Biloba (N06DP01) | 77(100) | *5* | 6.5 | *5* | 6.5 | *12* | 15.6 | *55* | 71.4 |
| Valerian (N05CP01/51) | 36(100) | *21* | 58.3 | *0* | 0.0 | *2* | 5.6 | *13* | 36.1 |
| St. Johns wort (N05CP03/N06AP01/51) | 13(100) | *7* | 53.8 | *1* | 7.7 | *1* | 7.7 | *4* | 30.8 |
| Other phytomedicines used for sleep | 7(100) | *6* | 85.7 | *1* | 14.3 | *0* | 0.0 | *0* | 0.0 |
| **All phytomedicines** | **133(100)** | ***39*** | **29.3** | ***7*** | **5.3** | ***15*** | **11.3** | ***72*** | **54.1** |
|  |  |  |  |  |  |  |  |  |  |
| **Total (all synthetics and all phytomedicines)** | **587(100)** | ***142*** | **24.2** | ***37*** | **6.3** | ***75*** | **12.8** | ***333*** | **56.7** |
|  |  |  |  |  |  |  |  |  |  |
| **Drug classes of interest** |  |  |  |  |  |  |  |  |  |
| Benzodiazepines and benzodiazepine-related drugs (N05BA, N05CD, N03AE01, N05CF) | 72(100) | *39* | 54.2 | *1* | 1.4 | *2* | 2.8 | *30* | 41.7 |
| Anti-depressants (N06A synthetics and St. John’s wort) | 140(100) | *16* | 11.4 | *6* | 4.3 | *22* | 15.7 | *96* | 68.6 |
| Anti-dementia drugs (N06D synthetics and Ginkgo biloba) | 84(100) | *5* | 6.0 | *7* | 8.3 | *14* | 16.7 | *58* | 69.0 |
| Hypnotics & sedatives (N05C synthetics, valerian and others) | 87(100) | *50* | 57.5 | *1* | 1.1 | *4* | 4.6 | *32* | 36.8 |
